# Kikuchi-Fujimoto Disease Following COVID-19

**DOI:** 10.7759/cureus.21049

**Published:** 2022-01-09

**Authors:** Hussain A Al Ghadeer, Sajjad M AlKadhem, Mohammed S AlMajed, Hassan M AlAmer, Jaber A AlHabeeb, Suad H Alomran, Abdullah S AlMajed

**Affiliations:** 1 Pediatrics, Maternity and Children Hospital, Al-Ahsa, SAU; 2 Pathology, Maternity and Children Hospital, Al-Ahsa, SAU; 3 Pediatrics, King Faisal University, Al-Ahsa, SAU

**Keywords:** novel coronavirus disease 2019., histiocytic necrotizing lymphadenitis, kikuchi-fujimoto disease, fever of unknown origin, cervical lymphadenopathy

## Abstract

Kikuchi-Fujimoto Disease (KFD) is a rare self-limiting condition of unknown etiology. It is characterized by fever, and lymphadenopathy most commonly involving posterior cervical lymph nodes. Although it is of uncertain etiology, it is associated with viral infections and autoimmune diseases. Distinction from lymphadenopathy-associated alternate disorders is crucial to avoid unneeded diagnostic procedures and treatment. KFD is diagnosed based on histopathologic examination of the excised lymph node. The management is supportive with favorable outcomes within a few weeks or months. In this case, we describe a 13-year-old boy who complained of painful cervical lymphadenopathy and fever for more than three weeks following COVID-19. Diagnostic workup has been established and KFD diagnosis made based on the histopathologic features of the involved lymph node. The patient showed complete recovery with no recurrence during follow-up. So, this case highlights the possible association between COVID-19 and KFD during this pandemic and keeping it in the differential diagnosis.

## Introduction

Kikuchi-Fujimoto disease (KFD), also known as histiocytic necrotizing lymphadenitis, is a rare self-limiting inflammatory disorder of unknown etiology, but viral or autoimmune causes may have a role. It is most commonly seen in Asia, predominantly affecting young women before the age of 30 years. Also, it can affect children with male predominance [1,2]. KFD is clinically characterized by a fever of unknown origin, painful cervical lymphadenopathy, and a cutaneous rash [3]. Although KFD is self-limiting and managed supportively, confounded with other diseases, including systemic lupus erythematosus (SLE), lymphoma can occur. The diagnostic tool is lymph node biopsy [4]. 

Children and adolescents with severe acute respiratory syndrome coronavirus 2 (SARS-CoV-2) infection experience mild COVID-19 with usually a low mortality rate. Cervical lymphadenopathy can be a clinical sign in these patients due to viral infection-induced reactive paracortical lymphoid hyperplasia. However, persistent and marked lymphadenopathy can be a clinical indication for fine-needle aspiration cytology (FNAC) or excision biopsy in these patients. Marked lymph node necrosis in the biopsy should prompt one to confirm or rule out KFD with the right index of suspicion. There are only three cases reported of Kikuchi-Fujimoto disease associated with SARS-CoV-2 infection [5-7]. In this case, we present a 13-year-old boy who presented with fever and lymphadenopathy following SARS-CoV-2 infection that presented with KFD.

## Case presentation

A previously healthy 13-year-old Saudi boy presented to the emergency department at Maternity and Children Hospital, Al-Ahsa, Saudi Arabia, complaining of neck swelling and fever for three weeks following a SARS-CoV-2 infection. Right-sided neck swelling was initially small and painless but, over time, became progressive and painful. There was no bleeding or discharge. He was initially evaluated by primary health care and treated with antibiotics (Augmentin) for two weeks with no improvement. This swelling was associated with intermittent high-grade fever reaching up to 39°C. He also was complaining of constitutional symptoms that included night sweating, weight loss due to anorexia, and on and off abdominal pain with generalized fatiguability. He denied any history of dysphagia, odynophagia, headache, change in bowel habits. There was no history of skin changes or joint pain. Other systemic reviews were unremarkable. One month prior to neck swelling, he was COVID-19 positive, treated conservatively with no intensive care unit (ICU) admission or hospitalization. There was a family history of lymphoma from the maternal side (mother, grandmother). Past surgical history revealed tonsillectomy a few years ago. He was fully vaccinated and did not have any type of allergy. There was no recent history of travel, contact with animals, or contact with tuberculosis patients.

The patient was looking well with no signs of distress during the clinical examination. Vitally, he was febrile with frequent spikes of fever during admission reaching up to 39.7°C with tachypnoea with a respiratory rate of 28 breath/minute, heart rate 120 beats/minute, and oxygen saturation 97% at room air. Local examination revealed cervical lymphadenopathy involving the posterior group of size 9*5 cm, mobile, discrete, tender on palpation with no inflammatory changes in the overlying skin or active discharge. Abdominal examination showed a soft, lax abdomen with no tenderness or organomegaly. Other systemic examinations had no abnormality was detected.

**Figure 1:**
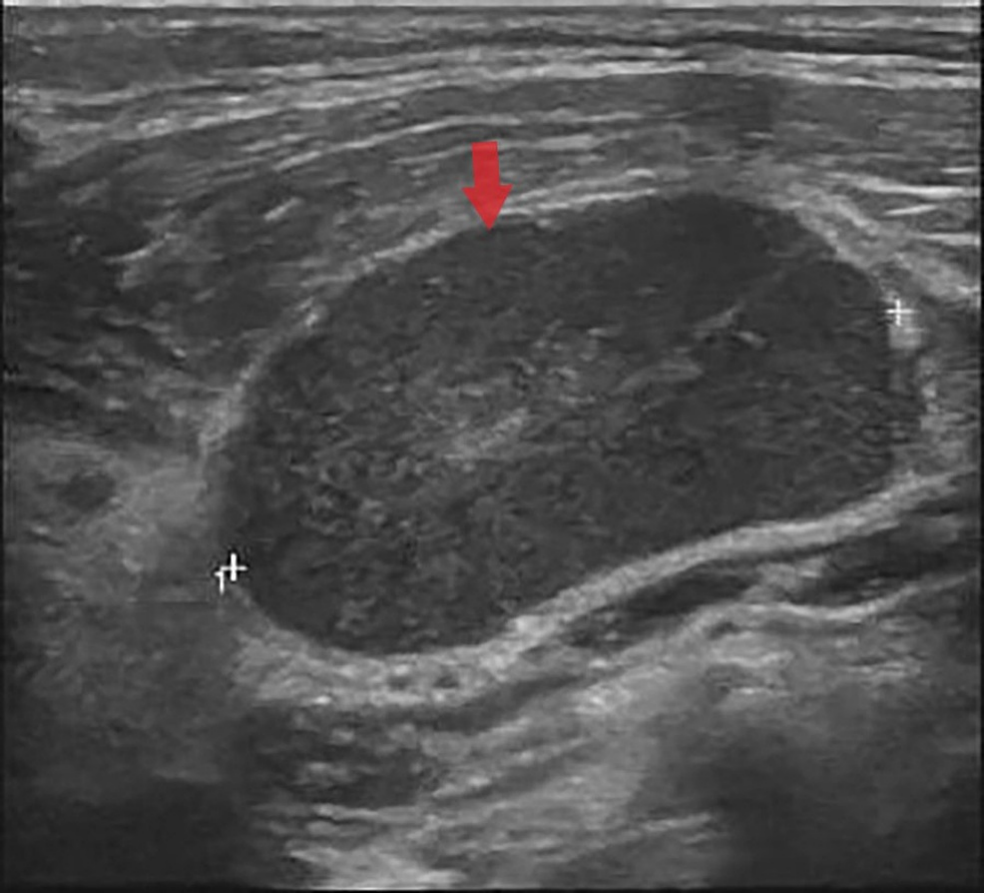
Homogeneous hypoechoic enlarged nodes with echogenic hilum

**Figure 2:**
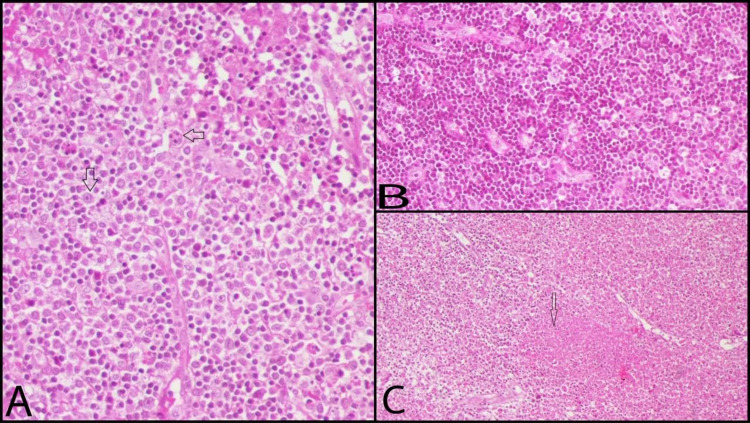
Findings of an excisional lymph node biopsy A: a high-power view (H&E X40) of a lymph node showing numerous histiocytes (down arrow) and scattered apoptotic cells (left arrow). There is no neutrophils seen. B: showing of a collection of histiocytes with a starry sky appearance. C: low power view of the lymph node with extensive necrosis (H X20).

Initial workup was established with imaging studies, serologies, and excisional biopsy of the lymph nodes. Laboratory investigations (Table 1) revealed leukopenia with absolute neutropenia and hypochromic microcytic anemia in complete blood count (CBC). Inflammatory markers, especially erythrocyte sedimentation rate (ERS) and lactate dehydrogenase (LDH) was high at 60 mm/hour and 538 units/L, respectively. Other investigations were unremarkable, including imaging studies. Neck ultrasound (Figure 1) showed multiple enlarged right non-necrotic lymph nodes at level I-V where the largest was seen at the level of V, about 2.8 x 1.7 cm. An excisional biopsy was carried out on the suspicion of a malignant lymphadenopathy (Figure 2)

**Table 1:** Laboratory investigations

Table 1: laboratory investigations		
Laboratory	Patient result	Reference range
Complete blood count (CBC)		
White blood cells	2.8	4.5-13.5 ×10^3^/µL
Neutrophil count	0.82	1.5-8.5 ×10^3^/µL
Lymphocyte count	1.73	1.5-6.5 ×10^3^/µL
Hemoglobin	11.3	12.5-13.7 g/dL
Platelets	179	150-350 ×10^3^/µL
Mean corpuscular volume (MCV)	69.5	77-86 fL
Renal profile		
Creatinine	70.92	44-88 µmol/L
Urea	2.99	1.7-7.1 mmol/L
Calcium	2	2.1-2.5 mmol/L
Sodium	138	135-145 mmol/L
Potassium	3.5	3.5-5 mmol/L
Chloride	103	97-107 mmol/L
Magnesium	0.70	0.63-1.05 mmol/L
Liver profile		
Aspartate aminotransferase (AST)	24	13-35 unit/L
Alanine aminotransferase (ALT)	21	10-30 unit/L
Alkaline phosphatase	82	100-320 units/L
Total serum bilirubin	0.42	0.2-1.5mg/dL
Direct bilirubin	0.18	0.0-0.2 mg/dL
Albumin	37	34.0-50.0 g/L
Lactate dehydrogenase (LDH)	538	100-190 units/L
Coagulation profile		
Prothrombin time (PT)	13.8	11.0-13.5 seconds
Partial thromboplastin time (PTT)	34.7	30-40 seconds
International normalized ratio (INR)	1.05	0.8-1.1
Inflammatory markers		
Erythrocyte sedimentation rate (ESR)	60	0.0-20 mm/hour
C-reactive protein (CRP)	Not available	
Cultures, serology results		
COVID-19 polymerase chain reaction (PCR)	Positive	
Blood culture	Negative	
Urine culture	Negative	
Cytomegalovirus antibodies	Negative	
Epstein-Barr virus antibodies	Negative	
Herpes simplex virus antibodies	Negative	
Purified protein derivative (PPD)	Negative	
Anti- Antinuclear antibodies (ANA)	Negative	
Anti-double stranded DNA (dsDNA)	Negative	
Rheumatoid factor	Negative	

The histological analysis demonstrated extensive necrosis, karyorrhexis, and ﬁbrin deposition with an absence of neutrophils. Flow cytometry revealed a reactive population of B lymphocytes and T lymphocytes. T lymphocytes were CD8+ positive, and the B lymphocytes were reactive and polyclonal. Echocardiography was normal. The above histopathological findings, clinical presentation, and other ancillary investigations were in keeping with a diagnosis of histiocytic necrotizing lymphadenitis.

The patient was treated conservatively with nonsteroidal anti-inflammatory drugs (NSAIDs), intravenous fluid, and regular monitoring. After five days, he became afebrile, and his general condition improved. Lymphadenopathy started to resolve, and the patient was discharged with analgesia as needed. During subsequent follow-up in the outpatient clinic, he was well with no recurrence of swelling or fever.

## Discussion

Since the World Health Organization stated that coronavirus disease 2019 (COVID-19) is a global pandemic, several associations with this virus have become clinically apparent, and works of literature and insight of this virus are expanding daily for comprehensive knowledge about it. SARS-CoV-2 has been implicated with inflammatory conditions, including multisystem inflammatory syndrome in children (MIS-C) and immunological complications including macrophage activation syndrome and cytokine storm syndrome [8,9]. This case highlights the possible association between KFD and COVD-19, although no conclusive evidence of COVID- 19 is a trigger for KFD. In 1972, Kikuchi-Fujimoto disease was described in Japan by Kikuchi and Fujimoto. It is also called histiocytic necrotizing lymphadenitis [10,11]. KFD is a benign self-limited disease with unknown etiology. Viral infections (Epstein-Barr virus, cytomegalovirus, rhinovirus, rubella virus, and HIV) and autoimmune diseases (systemic lupus erythematosus, polymyositis, rheumatoid arthritis, Still’s disease, and Sjogren’s syndrome) propose to be predisposing factors for KFD [4].

KFD causes immunological reactions that induced by T-cells to various antigens in genetically predisposed individuals. Human leukocyte antigen (HLA) class II alleles, particularly HLADPA1 and HLA-DPB1, have been discovered in most of the patients with KFD. These alleles are commonly seen in the Asian population in which makes them at higher risk of developing KFD other than non-Asian [12,13]. The clinical manifestations of KFD are lymphadenopathy, most commonly involving the posterior cervical node that is associated with pain, fever, and a skin rash. Joint involvement, hepatosplenomegaly, and constitutional symptoms are less frequently seen [2]. A patient with KFD complaining of weight loss, arthralgia, and exhibits skin manifestation with positive serologies is at higher risk of developing systemic lupus erythematosus (SLE) later on [14]. In this current case, the patient manifested with typical features of KFD with constitutional symptoms and mild hepatomegaly on abdominal ultrasound.

Lymphadenopathy is frequently seen in the pediatric age group. For this reason, KFD is usually misdiagnosed or mistaken with other lymphadenopathy-associated disorders, including malignancy, autoimmune diseases, and reactive lymphadenopathy due to viral infections [4]. A study reported that 30% of KFD cases were diagnosed incorrectly as lymphoma [15]. Therefore, early and accurate diagnosis with proper treatment is fundamental in KFD to avoid unnecessary procedures and treatment. Because there is no specific laboratory test or imaging study for KFD, the diagnosis can be made only by histological examination of the involved lymph node. The histological finding is characterized by non-caseating necrotic area, karyorrhectic nuclear debris surrounded by mononuclear cells, particularly CD68+ histiocytes, CD123+ plasmacytoid dendritic cells, and activated CD8+ T-lymphocytes [16]. The frequent laboratory findings are an elevated level of inflammatory markers (ESR and C-reactive protein [CRP]) and LDH. In a complete blood count (CBC), some studies revealed lymphopenia, thrombocytopenia, or leukocytosis [2,4]. In this particular case, there was revealed leukopenia with absolute neutropenia and hypochromic microcytic anemia in CBC with increased inflammatory markers. SLE was ruled out by negative anti-ANA, anti-dsDNA, and other viral-related lymphadenopathy were excluded. Due to the high index of suspicion of lymphoma, an excisional biopsy of the involved node revealed features of KFD.

There is no specific treatment known for KF; the majority of the cases are managed through supportive therapy for symptomatic relief. NSAIDs showed a good response. Systemic corticosteroids, hydroxychloroquine, or immunoglobulin are used in a severe form of KFD [2,17]. Complete resolution occurs within one to six months with a recurrence rate of 12.2% in children [1]. KFD leading to death is extremely rare (2.1%), usually due to systemic forms [18]. KFD can coexist, antedate or postdate with SLE, which has been reported in up to 25% of KFD cases [19]. So, long-term follow-up is essential for early recognition of SLE. Our patient was treated supportively, and general improvement was noticed within five days. A regular follow-up did not reveal a recurrence of lymphadenopathy or SLE. However, currently, the patient will be kept on long-term monitoring for SLE.

## Conclusions

KFD is a self-limited disorder characterized by fever and lymphadenopathy of unknown etiology. KFD should be differentiated from other serious lymphadenopathy-associated diseases include malignancy, autoimmune, inflammatory diseases, and viral infection. Thus, early lymph node biopsy is required for diagnosis to prevent unwarranted investigations and harmful treatment.

There are few cases reported about KFD associated with SARS-CoV-2 infection. The clinician should have a high index of suspicion when approaching a patient with unexplained lymphadenopathy following a SARS-CoV-2 infection.
